# Recombinant Acylation Stimulating Protein Administration to C3^−/−^ Mice Increases Insulin Resistance via Adipocyte Inflammatory Mechanisms

**DOI:** 10.1371/journal.pone.0046883

**Published:** 2012-10-08

**Authors:** Mercedes Nancy Munkonda, Marc Lapointe, Pierre Miegueu, Christian Roy, Danny Gauvreau, Denis Richard, Katherine Cianflone

**Affiliations:** Centre de Recherche Institut Universitaire de Cardiologie & Pneumologie de Québec, Université Laval, Québec, Canada; Beth Israel Deaconess Medical Center, United States of America

## Abstract

**Background:**

Complement 3 (C3), a key component of the innate immune system, is involved in early inflammatory responses. Acylation stimulating protein (ASP; aka C3adesArg), a C3 cleavage product, is produced in adipose tissue and stimulates lipid storage. We hypothesized that, depending on the diet, chronic ASP administration in C3^−/−^ mice would affect lipid metabolism and insulin sensitivity via an adaptive adipose tissue inflammatory response.

**Methodology/Principal Findings:**

C3^−/−^ mice on normal low fat diet (ND) or high fat diet (HFD) were chronically administered recombinant ASP (rASP) for 25 days via an osmotic mini-pump. While there was no effect on food intake, there was a decrease in activity, with a relative increase in adipose tissue weight on ND, and a shift in adipocyte size distribution. While rASP administration to C3^−/−^ mice on a ND increased insulin sensitivity, on a HFD, rASP administration had the opposite effect. Specifically, rASP administration in C3^−/−^ HFD mice resulted in decreased gene expression of IRS1, GLUT4, SREBF1 and NFκB in muscle, and decreased C5L2 but increased JNK, CD36, CD11c, CCR2 and NFκB gene expression in adipose tissue as well as increased secretion of proinflammatory cytokines (Rantes, KC, MCP-1, IL-6 and G-CSF). In adipose tissue, although IRS1 and GLUT4 mRNA were unchanged, insulin response was reduced.

**Conclusion:**

The effects of chronic rASP administration are tissue and diet specific, rASP administration enhances the HFD induced inflammatory response leading to an insulin-resistant state. These results suggest that, in humans, the increased plasma ASP associated with obesity and cardiovascular disease could be an additional factor directly contributing to development of metabolic syndrome, insulin resistance and diabetes.

## Introduction

The chronic inflammation observed in obesity is a key factor in the development of metabolic disorders leading to insulin resistance (IR) and diabetes. The contribution of adipose tissue to the overlap between metabolic and immune axes has become a subject of interest. A state of low-grade inflammation in obesity is associated with disturbances of adipose tissue-derived signaling molecules including adipokines such as acylation stimulating protein (ASP), adiponectin, leptin, as well as various cytokines/chemokines including interleukin-6 (IL-6), monocyte chemotactic protein-1 (MCP-1), Granulocyte colony-stimulating factor (G-CSF), keratinocyte-derived chemokine (KC) and Rantes [1], [2], [3], [4], [5], [6]. At the tissue level, some of these adipocytokines (e.g., IL-6, KC and MCP-1) induce inflammatory pathways that lead to accumulation and activation of neutrophils, monocytes and macrophages and promote both positive and negative responses [6], [7], [8], [9], [10]. Accumulating evidence pinpoints adipose tissue macrophage (ATM) accumulation as a critical factor in proinflammatory cytokine production that promotes obesity-induced tissue inflammation. Two specific macrophage subpopulations are at the forefront: F4/80^+^/CD11b^+^/CD11c^+^, M1-like macrophages indicating proinflammatory responses in diet induced obesity (DIO); and, F4/80^+^/CD11b^+^/CD11c^−^, M2-like macrophages which maintain homeostasis by suppressing proinflammatory signals [10], [11], [12].

Numerous recent studies indicate that chronic increases in circulating inflammatory cytokines, adipokines as well as monocytes are associated with inflammation-related changes in adipose tissue leading to IR [8], [9]. Through immune cell infiltration in adipose tissue, muscle or liver, these endogenous factors are involved in reduction of both systemic and local insulin sensitivity [8], [9]. Further, targeted genetic deletions in adipose tissue or macrophages of inflammatory pathway components such as nuclear factor-κB (NF-κB) and C-Jun-terminal kinase (JNK) protected against diet-induced IR and glucose intolerance while, by contrast, deletion of macrophage-specific PPARγ enhanced IR [13], [14], [15]. JNK and NFκB mRNA expression are elevated in obesity, mediated by free fatty acids and inflammatory cytokines, and this has been implicated in the development of diabetes [13], [15]. However, the mechanisms underlying initial inflammatory cell recruitment and activation are not completely understood.

From an immune perspective, innate immune component complement 3 (C3) and its mediator molecules, generated during C3 activation and cleavage (e.g., C3a, C5a, C4b, C3b), play critical roles in inflammation [16], [17], [18]. C3-derived anaphylatoxins C3a and C5a act through C3a and C5a receptors (C3aR and C5aR, respectively), to promote initial infiltration of inflammatory cells in tissue sites such as liver, lung and kidney [17], [19]. However, the role of C3 and its products (including ASP, aka C3adesArg) in adipose tissue macrophage infiltration is unknown. While C3aR is present in adipose tissue, expression of C5aR is unknown [19]. It is known that ASP does not bind C3aR and C5aR, but its binding to C5L2 remains controversial; this receptor has also been proposed as a C5a decoy receptor, decreasing chemotactic responses by preventing C5a binding to C5aR [17], [22], [23]. ASP, C3a and C5a are all ligands for C5a-like receptor 2 (C5L2) receptor [20], [21]. C5L2 mRNA expression was identified in brain, adipose tissue, muscle, liver and immune cells [22], [24]. Many of these studies have used transfected cells to evaluate C5L2 expression and function, however there are a few studies on endogenously expressing cells. Bamberg and colleagues [25] demonstrated that in human PMNs, C5L2 is primarily (60–80%) intracellular. Nonetheless, cell surface C5L2 has been demonstrated in HL-60, HeLa, neutrophils, PMNs, human fibroblasts, preadipocytes, cultured adipocytes and primary adipocytes, although the intracellular component was not evaluated [26], [27], [28], [20].

Moreover, we have previously shown that C5L2 is a bioactive receptor for ASP [20], [21]. ASP response requires the presence of C5L2, and antisense downregulation of C5L2 decreases ASP responsiveness [29]. The metabolic profile of C5L2^−/−^ mice resembles C3^−/−^ mice (which are obligately ASP deficient mice), and adipose tissue from C5L2^−/−^ mice does not respond to ASP stimulation [30], [20]. ASP (C3adesArg) is produced within adipose tissue through cleavage of the precursor protein complement C3 via factor B and adipsin (factor D) interaction, generating C3a, which is rapidly desarginated by carboxypeptidase B to produce C3adesArg [31]. *In vitro*, ASP has potent lipogenic activity, stimulating fatty acid uptake, triglyceride (TG) synthesis and storage, and glucose uptake as well as inhibiting lipolysis in adipocytes via interaction with C5L2 [31], [32]. *In vivo*, acute administration of ASP in wildtype and obese mice accelerates postprandial TG clearance [31]. Chronic ASP administration in wildtype mice on a high fat diet leads to decreased energy expenditure, increased body weight and food intake and decreased liver glycogen [33].

However, it is not clear if these effects obtained are caused solely by the increased endogenous ASP (due to the HFD, [34]) or the combination of high endogenous ASP + surplus administered ASP. It is also unknown if these changes directly affect insulin response and inflammation. As ASP, and its carboxy-terminal peptides do not bind or activate C3aR [35], [36], ASP has usually been considered an inactive immune by-product [18], [31], [32]. However, several studies suggest that ASP may mediate additional functions beyond fatty acid storage in adipocytes. Specifically, ASP induces glucose-stimulated insulin release from pancreatic cells; modulates cytokine synthesis by human peripheral blood mononuclear cells (PBMC, [16] and tonsil-derived B cells [37]) as well as inhibits cytotoxicity of natural killer (NK) cells [38].

In humans, plasma ASP levels in non-obese people range from 10–58 nmol/L and are increased 25% to 4-fold in obesity, cardiovascular disease and diabetes [31]. However, obesity is not an essential feature of elevated ASP levels, as ASP is also increased in subjects with type 2 diabetes, polycystic ovary disease, and lipoprotein lipase deficiency even in the absence of obesity [31], [39], [40], [41]. In mice, reported plasma ASP levels range from 3–12 nmol/L, increasing with HFD and in obese mouse models, and decreasing in genetically lean mice [34]. Very few studies have evaluated the involvement of ASP/C3adesArg in inflammatory pathways and immune-adipose interaction [42], [16], [37], [43]. For example, some studies have shown a correlation between circulating ASP and IL-6 levels [33], [44]. Therefore it is unknown whether the presence of increased ASP in these circumstances could directly contribute to the negative effects of a high caloric diet.

The *in vivo* role of ASP has been studied in C3^−/−^ knockout mice (C3KO), which cannot produce either ASP or C3a [45]. This knockout mouse model has shown an obesity-resistant phenotype, with delays in postprandial TG and non-esterified fatty acid (NEFA) clearance [34]. In addition, C3KO mice have decreased fat storage through increased energy expenditure [34]. In the C3^−/−^ model (deficient in ASP, C3a and C5a), acute bolus administration of ASP reconstitutes the ASP-C5L2 pathway, but as ASP binds neither to C3aR or C5aR, this bolus administration avoids the potential effects mediated through C3a-C3aR and C5a-C5aR, both recognized to be involved in immune cell recruitment in the initial inflammation phase. In the present study, a direct evaluation of the diet-permissive role of ASP in systemic, adipose tissue, muscle and liver IR-related inflammation was evaluated through chronic administration of recombinant ASP with a normal low fat diet (ND) or a high fat diet (HFD) for 3 weeks.

## Results

### ASP Alters Energy Usage and Energy Storage

**Figure 1:**
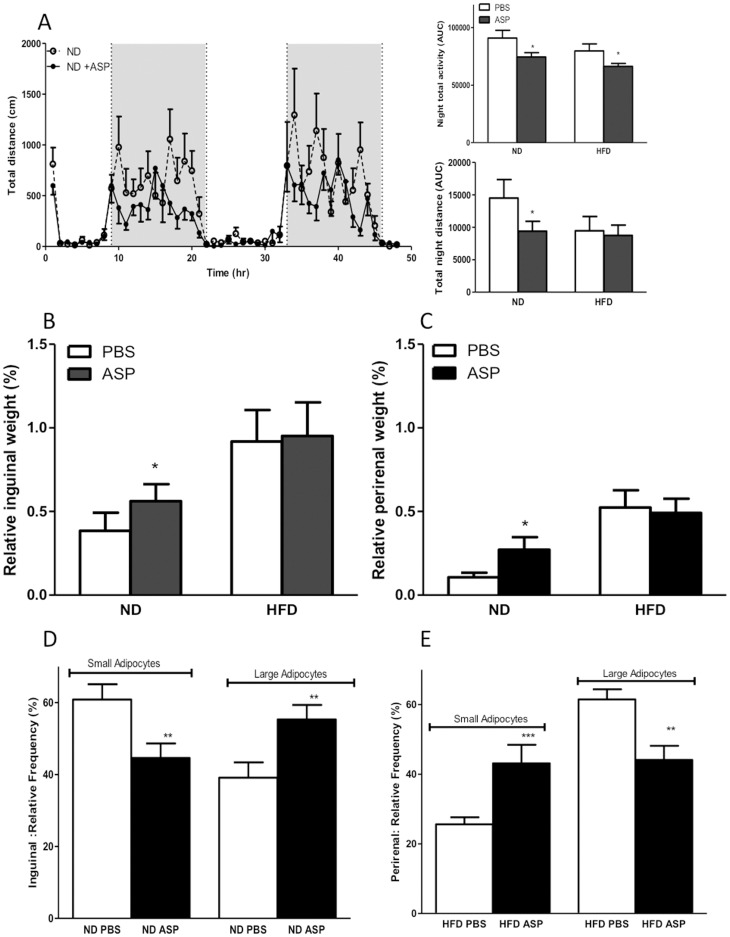
rASP administration alters total activity and adipose tissue. A) Activity of C3KO mice on ND ±ASP administration measured over a 48 hour period (dark cycles are indicated as shaded areas). **B and C)** Relative adipose tissue mass (percent of total body weight) of C3KO or C3KO+ ASP mice fed on ND and HFD. **D and E)** Distribution of small and large adipocytes in adipose tissues of C3KO or C3KO+ ASP mice fed on ND and HFD. Results are presented as average ±SEM for n = 9−10 where *p<0.05, **p<0.01, ***p<0.001.

Male C3KO mice were fed with normal diet (ND, 10% fat) or high fat diet (HFD, 45% fat) for 1 week prior to ASP administration by mini pump for 3 weeks. The final average circulating recombinant levels of ASP that were detected in the mouse plasma were 8.0 nM, within normal ranges for mice (2.3 nM ND, 9.7 nM HFD, [34]. The results show that there was no difference in food intake and total energy expenditure (VO_2_, VCO_2_) with/without ASP administration under ND or HFD feeding conditions over 4 weeks (data not shown). However, there was an ASP-induced decrease in activity (total night horizontal distance and total activity) on both ND and HFD-fed mice **(** **Figure 1A** **, and inset)**.

On the other hand, while starting body weights were the same, C3KO mice with ASP administration gained more weight over the study (data not shown). This translated into a difference in adipose tissue mass with increased inguinal, (+19.6±21.6%) and perirenal mass, (+153.7±69.5%) in ND-fed C3KO+ASP *vs.* C3KO **(** **Figure 1B, C** **)**. HFD-fed mice also increased body weight and fat mass, but there was no additive effect of ASP over diet. Analysis of adipocyte size distribution indicates that in ND feeding, ASP administration reduces small-sized adipocytes and increases larger adipocytes **(** **Figure 1D** **)**. In high fat feeding, the size of the adipocytes overall was increased, and there was a greater increase in the quantity of smaller adipocytes **(** **Figure 1E** **)**. Together, these data suggest that ASP affects adipose tissue morphology differentially on normal diet compared to high fat diet.

We also measured plasma lipid parameters such as NEFA, triglyceride and cholesterol. Triglyceride and NEFA levels were unchanged under any feeding condition although the total cholesterol levels were increased in ND-fed C3KO in the presence of ASP **(** **Table 1** **)**.

**Table 1:** Plasma lipid parameters of C3KO and C3KO+ ASP mice.

	ND		HFD	
Parameters	C3KO	C3KO + ASP	C3KO	C3KO + ASP
Triglycerides (mmol/L)	0.45±0.02	0.46±0.03	0.55±0.07	0.43±0.03
NEFA (mmol/L)	0.38±0.02	0.46±0.04	0.44±0.02	0.38±0.02
Cholesterol (mmol/L)	1.65±0.10	1.97±0.10*	2.05±0.15	1.91±0.12

C3KO mice were fed on ND or HFD for 1 week before ASP was added by mini pump for 3 weeks more, and plasma was collected after 6h fast (n = 9−10 mice per group).

* Indicates a statistical difference within a dietary treatment group (p<0.05).The data presented are Mean ± SEM

### ASP Gain-of-function Altered Adipose Tissue and Muscle Insulin Sensitivity Differentially

**Figure 2:**
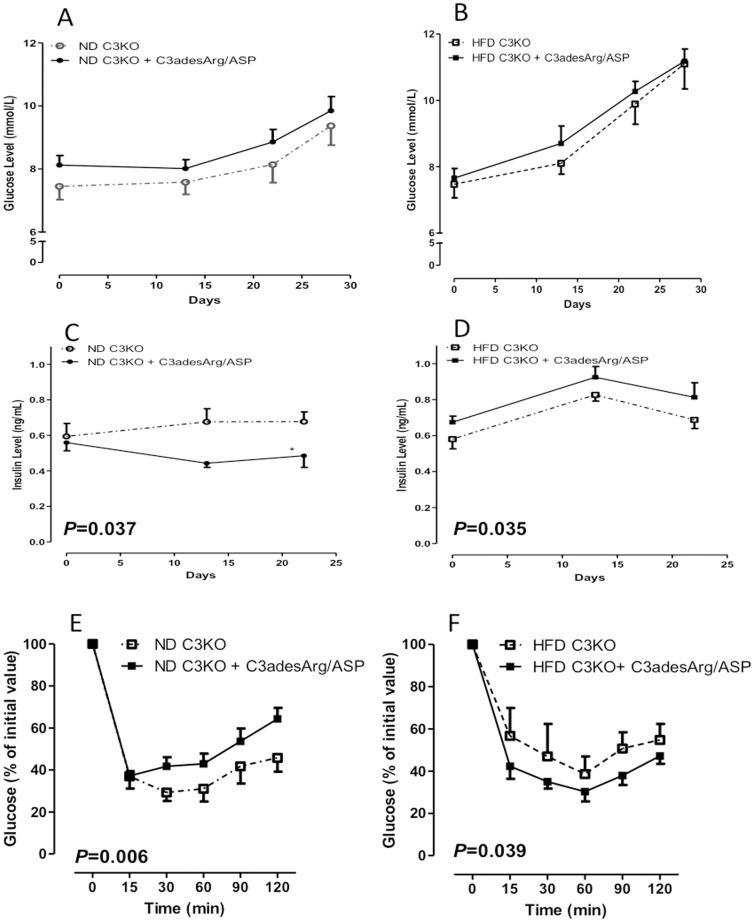
Chronic 4-week ASP treatment altered C3KO peripheral insulin sensitivity. Results are presented for glucose and insulin levels over the time course of treatment for ND (**A, C**) and HFD (**B, D**). **E and F)** Insulin tolerance tests in C3KO mice ±ASP on ND (E) and HFD (F) Results are presented as average ±SEM for n = 8−9 where values for 2way ANOVA are provided in graphs.

Fasting glucose and insulin were evaluated throughout the study, in addition, an insulin tolerance test (ITT) was performed in ND and HFD fed mice. The results indicate that chronic ASP administration had no effect on fasting blood glucose levels in ND and HFD fed mice **(** **Figure 2A and B** **)**. However, in presence of ASP, fasting insulin levels decreased in ND C3KO **(** **Figure 2C** **)**. This was further supported by ITT test results, which showed that the ND-fed mice treated with ASP had improved insulin sensitivity with post-ITT glucose levels returning towards baseline more rapidly **(** **Figure 2E**). By contrast, compared to HFD-fed C3KO mice, HFD fed-C3KO+ASP mice had increased fasting insulin levels (**Figure 2D**), suggesting an onset of systemic insulin resistance, which was further supported by ITT, where mice administered with ASP had a greater delay in return to fasting glucose levels **(** **Figure 2F** **)**.

**Figure 3:**
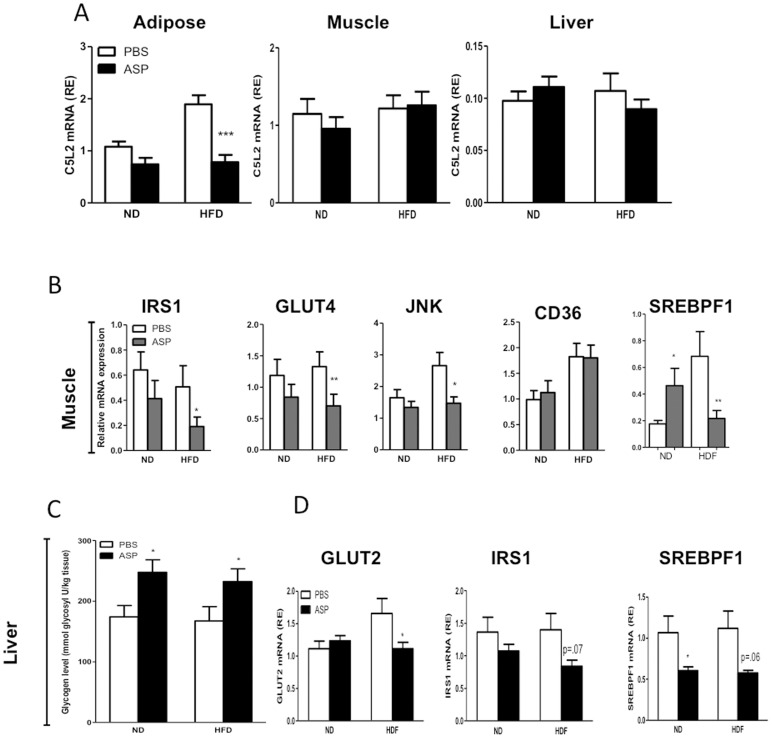
ASP effects on mRNA expression of C5L2 and Insulin-related genes adipose tissue, muscle and liver. A) C5L2 mRNA gene expression in perirenal (left panel), muscle (center panel) and liver (right panel) tissues. **B)** Gene expression in muscle tissue, **C)** Liver glycogen levels and **D)** liver gene expression levels from C3KO or C3KO+ASP mice fed on ND and HFD. Results are presented as average ±SEM (n = 5−8), where *p<0.05, **p<0.01 and ***p<0.001.

Because adipose tissue, muscle and liver are all major insulin-sensitive tissues that contribute to systemic insulin resistance by modulating insulin action, gene expression of C5L2, the ASP receptor, in adipose tissue, muscle and liver was evaluated. As shown in **Figure 3A**, a high fat diet resulted in increased C5L2 mRNA expression in C3KO adipose tissue. However, after chronic ASP administration, C5L2 mRNA was down-regulated in adipose tissue. Neither ASP nor high fat diet had any effect on C5L2 mRNA expression in muscle or liver **(** **Figure 3A** **)**.

In muscle tissue of high fat fed mice, decreases in IRS1, GLUT4 and JNK mRNA expression were evident with ASP-administration **(** **Figure 3B** **)**. SREBPF1 mRNA was increased in ND but decreased in HFD. However, ASP had no effect on muscle genes involved in lipid metabolism (mRNA expression of CD36 (Figure 3B) ACACA (not shown), or on muscle tissue triglyceride or NEFA levels (not shown).

**Figure 4:**
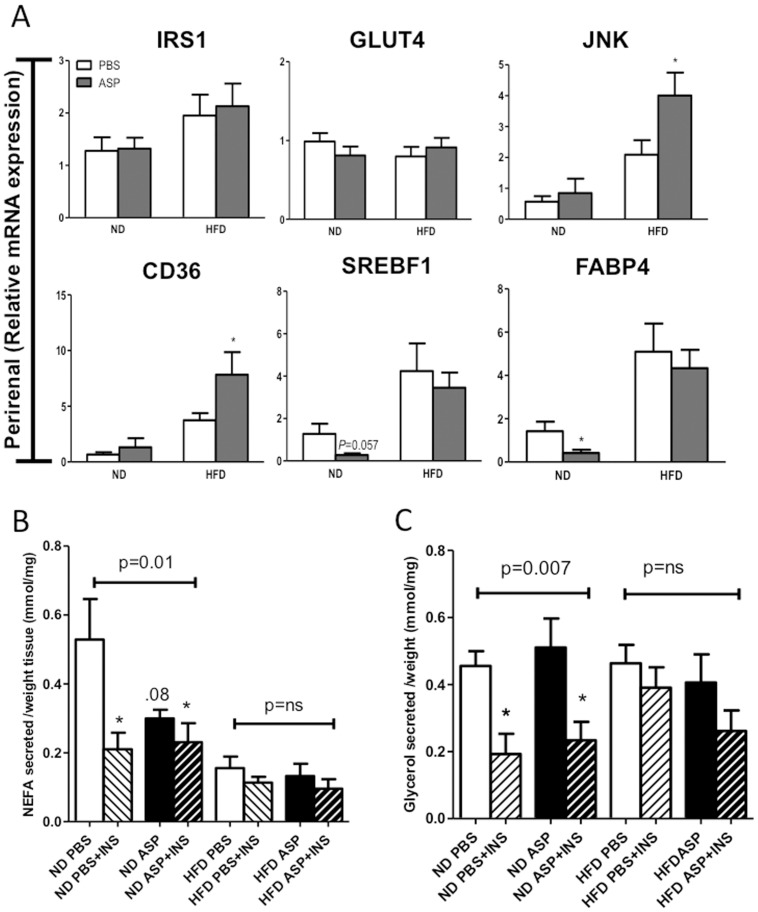
ASP effects on mRNA expression of insulin-related genes, and insulin lipolytic function in adipose tissue. A) Gene expression in perirenal adipose tissue, **B and C)** Lipolysis measured as non-esterified fatty acid release (B) or glycerol release (C) in perirenal adipose tissue from C3KO mice ±ASP treatment on ND and HFD, followed by ex vivo stimulation ± insulin for 3 hours. Results are presented as average ±SEM (n = 5−8), where *p<0.05, **p<0.01 and ***p<0.001.

In liver, both ND and HFD mice treated with ASP demonstrated increased glycogen levels **(** **Figure 3C** **)**, however there was no effect on tissue triglyceride or NEFA levels (not shown). In HFD, there were significant ASP-induced changes in GLUT2, IRS1, and SREBPF1 gene expression **(** **Figure 3D** **)**, but no change in FABP4 or JNK (data not shown). In contrast to muscle, adipose tissue of ND or HFD-fed mice receiving ASP showed no ASP-mediated change in GLUT4 or IRS1 while JNK and CD36 levels increased in HFD **(** **Figure 4A** **)**. Furthermore, ASP administration in ND-fed mice had a tendency to decrease mRNA expression of SREBPF1 and FABP4 **(** **Figure 4A** **)**. Inguinal (not shown) and gonadal (**Figure 4B and C**) adipose tissues retained their insulin responsiveness in mice receiving ASP, based on insulin inhibition of fatty acid (NEFA) and glycerol release, although the insulin response was blunted with HFD **(NS,** **Figure 4B and C** **)**. Together, these data suggest that ASP administration induced alterations in adipose, muscle and liver lipid metabolism and/or insulin sensitivity and these disturbances were closely linked to the nutrient state.

### HF-fed C3KO+ASP Administration Increases Inflammatory Adipose Tissue Profile

**Figure 5:**
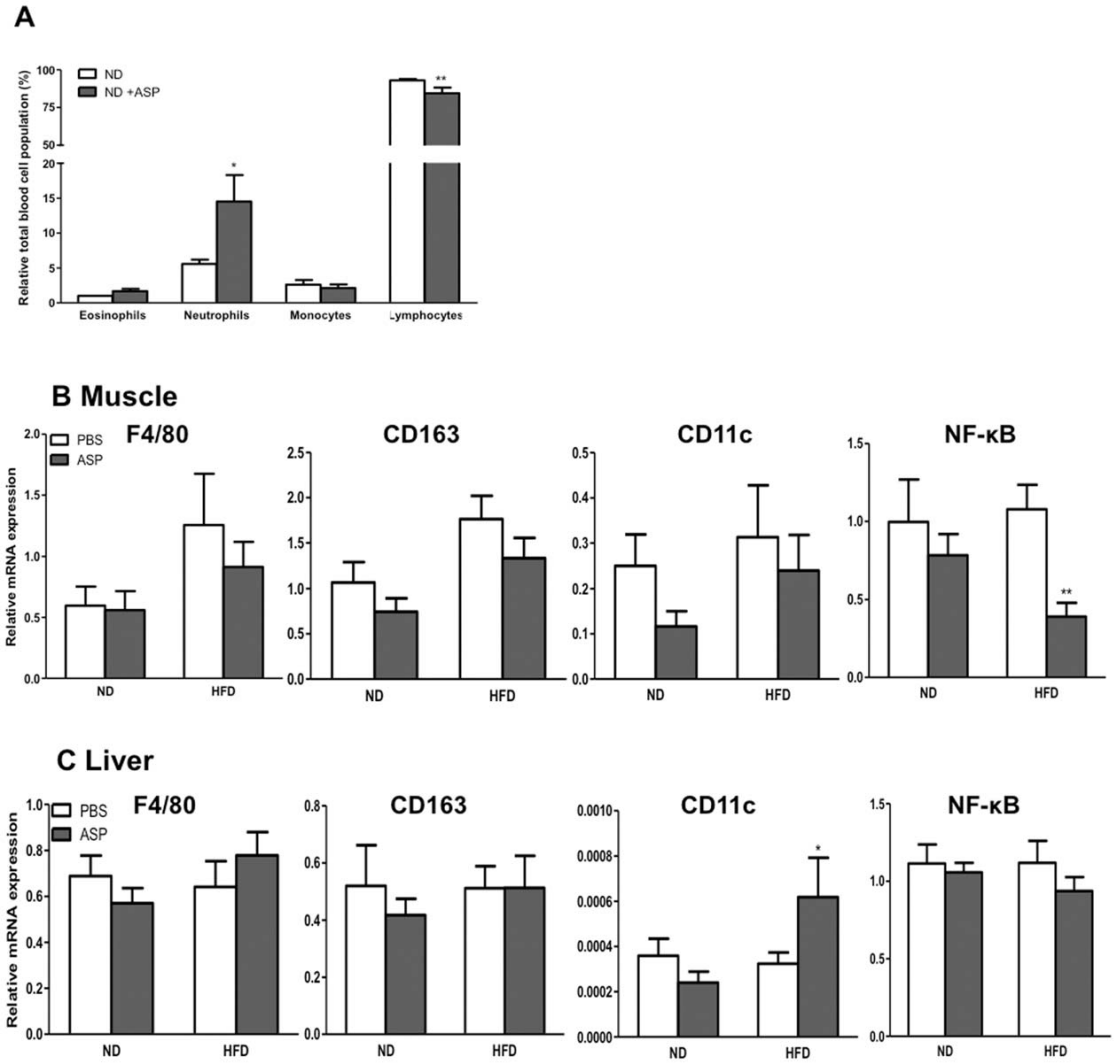
ASP effects on circulating immune cells and mRNA of immune-related genes in muscle and liver. A) Circulating immune cell distribution in C3KO ±ASP administration on ND. **B and C)** Gene expression in muscle (B) and liver (C) of immune-related genes in C3KO ±ASP on ND and HFD. Results are presented as average ±SEM (n = 5–8), where *p<0.05, **p<0.01 and ***p<0.001.

We evaluated the effects of ASP treatment on the inflammatory profile of adipose tissue, muscle and liver using RT-qPCR. First, as shown in **Figure 5A**, there was a change in circulating immune cell distribution following ASP administration, with an increase in neutrophils and a decrease in lymphocytes on normal low fat diet feeding. There was no difference in blood immune cell distribution on HF-fed mice with ASP administration (data not shown). However, with high fat feeding, in muscle, ASP administration induced decreases in NF-κB, with no significant changes in F4/80, CD163, or CD11c **(** **Figure 5B** **)**. In liver, mRNA expression of NF-κB, as well as F4/80 and CD163 were not changed while mRNA expression of CD11c, while weakly expressed, was increased **(** **Figure 5C** **)**.

**Figure 6:**
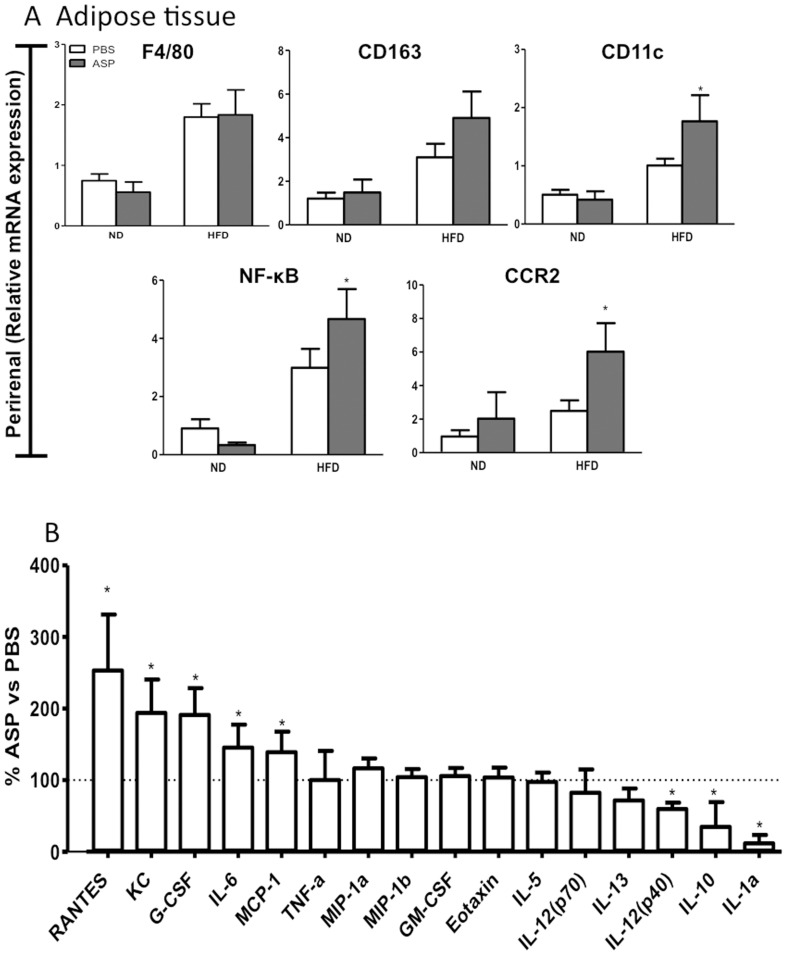
ASP administration induces changes in adipose tissue cytokine secretion and immune-related gene expression. A) Gene expression in perirenal adipose tissue in C3KO ±ASP in ND and HFD mice. **B)** Cytokine secretion in ex vivo adipose tissue from C3KO or C3KO+ASP mice fed on ND and HFD incubated for 24 hours (n = 6–9). Results are presented as average ±SEM (n = 5–8), where *p<0.05, **p<0.01 and ***p<0.001.

In adipose tissue, mRNA expression of F4/80 was unchanged, but CD163, CD11c, NF-κB and CCR2 all increased with ASP treatment, especially in the HFD **(** **Figure 6A** **)**. The changes in inflammatory genes in adipose tissue were further supported by profile analysis of secreted adipocytokines from adipose tissue. As shown in **Figure 6B**, when taken together (inguinal & gonadal adipose tissues on ND and HFD) the effects of ASP on adipose tissue cytokine secretion demonstrated an increase in RANTES, KC, G-CSF, IL-6, and MCP1 with a decrease inIL-12, IL-10 and IL-1α. There were no ASP-mediated changes detected in media from liver incubations (data not shown).

## Discussion

The aim of the present study was to investigate the interaction between diet (low fat vs. high fat) and presence/absence of ASP on adipose tissue low-grade inflammation and on the development of insulin resistance using a C3^−/−^ mouse model (which is obligately ASP deficient) subjected to normal low fat and high fat fed settings. Previously, acute ASP administration had been evaluated in lean (C3KO and wild type) and obese (*ob*/*ob* and *db/db*) mice in other studies [31], [46]. These studies all showed that bolus administration of ASP enhanced postprandial triglyceride, NEFA and glucose clearance as well as increased adipose tissue fatty acid storage [31], [46]. Four weeks of osmotic mini-pump chronic ASP administration in wild type mice showed similar results to the acute bolus studies [33]. Important differences between the present and previous studies exist: none of the previous studies have directly investigated the diet-permissive effects of ASP in adipose tissue linked to low-grade inflammation or associated insulin resistance. Further, in the wildtype mice, as high fat diet increases endogenous ASP levels [34], we cannot evaluate the contribution of diet vs ASP effects, nor rule out that the effects are due to excessively high ASP (endogenous plus administered). In complementary studies, acute administration of bolus ASP in C3KO mice highlighted the role of ASP in the balance between inflammation/injury versus regeneration following partial liver resection [47]. In cats, bolus administration of ASP (C3adesArg) increased inflammation in the central nervous system [42]. Our present results demonstrated that ASP administration (at physiological levels) had diverse effects specific to diet as well as tissue. The most marked difference was the protection from insulin resistance on a low fat ND, but the exacerbation of insulin resistance on a HFD. In general, the ASP mediated changes were more pronounced on a HFD.

In ND feeding with ASP administration, treatment resulted in increased adipose tissue weight (inguinal and perirenal depots) with an increased number of larger adipocytes. There was no change in expression of C5L2, suggesting conservation of ASP sensitivity. However, ND-fed C3KO+ASP mice had little change in adipose tissue macrophage inflammatory pathway expression (F4/80, CD163, CD11c, JNK, NF-κB and CCR2 mRNA expression) but reduced adipose tissue lipid storage gene expression (SREBP1). Further, there was no change in circulating NEFA levels, nor any changes in circulating or tissue lipid content. In addition, glycogen depots increased in liver, suggesting increased liver glucose storage may contribute to maintaining blood glucose homeostasis. Notwithstanding the increased adipose tissue while on a normal low fat diet with ASP, overall ASP administration appeared to be protective of insulin sensitivity based on *in vivo* ITT, *ex vivo* insulin inhibition of lipolysis, and adipose, muscle and liver gene expression. Since adipose tissue, muscle and liver are key players in systemic insulin resistance, this data suggests that ASP, independently of its adiposity effects, influences insulin sensitivity in the absence of diet induced-obesity. This is in agreement with ASP additive effects on insulin response in both adipose tissue and muscle glucose transport as previously shown [31], [32].

By contrast, on a high fat diet, there is a diet-induced increase in adipose tissue, and ASP administration further increased the number of small adipocytes relative to large adipocytes suggesting adipose tissue hyperplasia as a compensatory mechanism. ASP administration induced an onset of low-grade chronic inflammation as can be seen by the increases in expression of inflammatory genes in adipose tissue (CD11c, JNK, NF-κB and CCR2) as well as in *ex vivo* secreted proinflammatory cytokines (RANTES, KC, G-CSF, IL-6 and MCP1) which act as chemoattractant factors for immune cell migration into adipose tissue [6], [7], [48]. These changes in proinflammatory profile coupled to the HFD may directly relate to the decreased insulin sensitivity in HFD C3KO + ASP mice, as demonstrated by decreases in tissue expression of IRS1, GLUT4, SREBPF1, GLUT2, decreased insulin-mediated inhibition of lipolysis in adipose tissue, increasing fasting circulating insulin levels, and changes in ITT profile.

Based on the changes induced by ASP administration, we can propose that proinflammatory adipocytokines activate several kinases such as JNK and NF-κB that, in turn, impair insulin signaling at the insulin receptor and insulin substrate receptor 1 (IRS1) level [13], [49]. By contrast, as shown elsewhere, an absence of JNK1 results in decreased adiposity and decreased cytokine production and secretion (such as IL-8 (KC) and G-CSF) [50], which significantly improves insulin sensitivity and enhances insulin receptor signaling capacity. Previously we showed that ASP and insulin activate the mitogen-activated protein kinase/extracellular signal-regulated kinase (MAPK/ERK) pathway [51]. Here, our data shows that ASP administration modulates NF-κB and JNK gene expression as well. JNK is one of the 3 members of the mitogen-activated protein kinase (MAPK) superfamily. In addition, the ASP-induced increase in proinflammatory cytokine secretion supports involvement of the ASP-C5L2 system in the recruitment of immune cells, particularly M1-like macrophages, into adipose tissue. Together, our data suggest a proximal role of ASP in HFD-induced adipose tissue low-grade inflammation.

**Figure 7:**
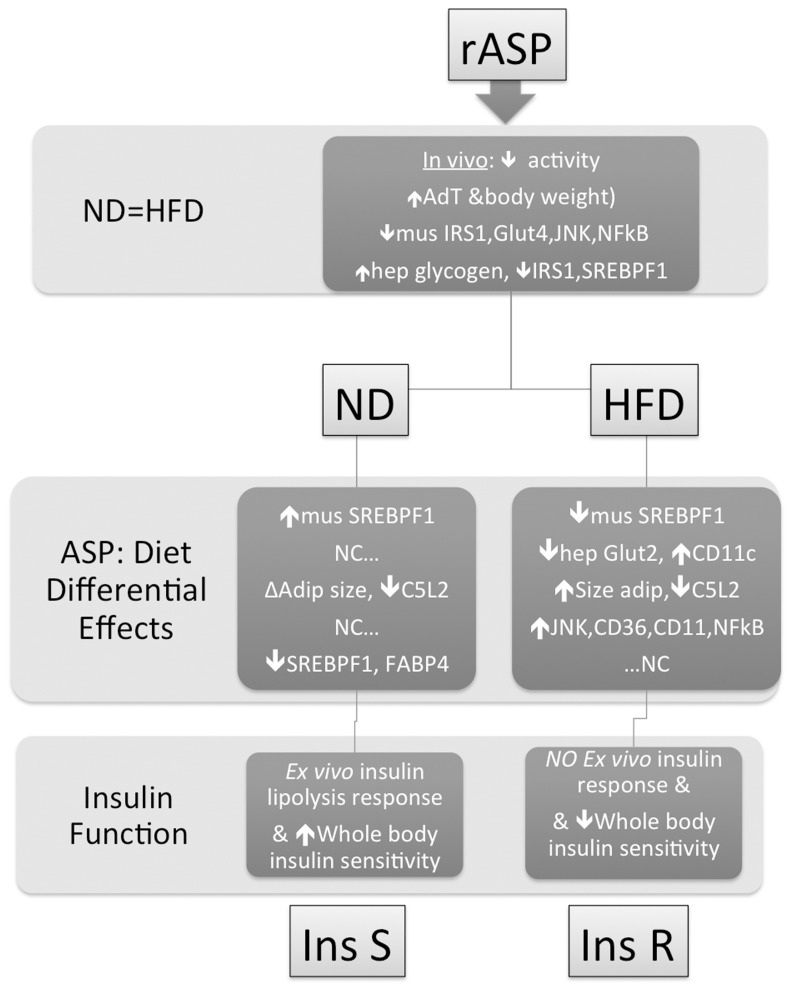
Diagram of low fat diet and high fat diet induced interactions with rASP administration. Recombinant ASP administration induced changes in adipose tissue (AdT), muscle (mus) and liver (hep) that, in some cases, were identical in both diets (ND = HFD panel). On the other hand, there were many differential changes according to the diet (normal low fat diet, ND, vs high fat diet, HFD) as outlined in the ASP: Diet Differential Effects panel. As a consequence, rASP administration led to enhanced insulin sensitivity in ND, but led to greater insulin resistance in HFD (Insulin Function panel), where NC = no change.

In summary, we show that ASP has a tissue-specific function, which is also influenced by nutrient (diet) availability. These diet-specific ASP induced effects are summarized graphically in Figure 7, demonstrating the key changes in muscle, liver and particularly adipose tissue that may explain the HFD permissive association with insulin resistance. During HFD feeding, ASP exacerbates adipose tissue low-grade chronic inflammation, which is linked to other obesity-derived metabolic disorders such as IR. Together, the results suggest that ASP contributes to the initiation of the inflammatory response in adipose tissue and demonstrates another mechanism by which C3 and its effector proteins (ASP) may regulate immune responses leading to insulin resistance in an obesogenic environment.

Based on these mice studies, we propose that in humans, the chronically increased ASP levels in various metabolic disturbances including obesity, diabetes, cardiovascular disease, polycystic ovary syndrome and lipoprotein lipase deficiencies [31], [40], [41], [39] may directly contribute not only to adipose tissue dysfunction, but to chronic inflammatory responses and insulin resistance, particularly in the presence of typical Western high fat diets.

## Materials and Methods

### Mice

Male and female C3KO mice purchased from Jackson Laboratories were bred in our animal facility. All protocols were conducted in accordance with the CACC guidelines and approved by the Centre de Recherche Institut Universitaire en Cardiologie et Pneumologie de Quebec and the Laval University Animal Care Committee. For this study, male mice were individually housed in a sterile-barrier facility under 12 h:12 h light-dark cycle. At 12 weeks, mice were placed on either standard normal low fat diet (ND, 10% kcal fat; Charles River Laboratories, Wilmington, PA, USA) or high fat diet (HFD, 45% kcal fat; Research Diets Inc., New Brunswick, NJ, USA) for 4 weeks. 10 days following the start of the HFD, the mice were surgically implanted with an osmotic mini-pump (model 2004, Durect Corp, Cupertino, CA, USA.). Briefly, the mice were first sedated with ketopiofen (5 mg/kg) then anesthetized with isofluorane. Under sterile conditions, a small incision was made between the skin and scapulae and the osmotic pump was inserted subcutaneously into the mouse. The incision was then closed with sutures according to manufacturer’s instructions. The mice received; recombinant ASP (rASP, 142 pmol/µL) or PBS (vehicle) via continuous delivery for 28 days at a flow rate of 0.25 µL/hr (n = 9–10 mice per group). Body weight and food intake were measured two to three times per week for 4 weeks. Calorimetric measurements were done 12–15 days after surgery. Oxygen consumption (VO_2_) and carbon dioxide production (VCO_2_) were measured over a 24 hour period in an open circuit system after 48 hours equilibration as previously described [33]. VO_2_ and VCO_2_ were calculated as mL/kg/min and RQ (respiratory quotient) was taken as the ratio of VCO_2_/VO_2_. Physical activity was determined by breaks in photo beams and converted into distance from the horizontal beam (cm/24-h period), and total activity was obtained from all of the beams (arbitrary units/day). On the twenty-fifth day, following mini-pump insertion (4 weeks on HFD), mice were fasted for 6 h, and then euthanized. Blood was then collected through cardiac puncture, and tissues and mini-pump were harvested and immediately frozen in liquid nitrogen, then transferred to −80°C.

### Purification of Recombinant ASP (rASP)

Recombinant human ASP (rASP) was produced and purified as previously described in detail; including sequential extraction, Ni^2+^ sepharose binding and HPLC reverse-phase chromatography [33] [20], [52]. Detailed characterization is provided elsewhere [20], however, HPLC-purified ASP preparations used in injections were >98% pure based on mass spectrometry, and were C5a, insulin and endotoxin negative. Following HPLC, rASP is reconstituted in a physiological buffer, PBS, and buffer alone is used as a negative control in injections.

### Plasma and Media Analysis

Blood was collected after 6 h of fasting. At time of dissection, minced pieces of adipose tissue and liver were incubated ex vivo for 18 h in tissue culture media (37°C, 5% CO_2_ incubator), media was collected and frozen at −80°C for later analysis. Plasma glucose levels were measured using a glucometer. Plasma triglyceride (TG), non-esterified fatty acid (NEFA) and cholesterol were measured by colorimetric enzymatic kits as follows: plasma TG (Roche Diagnostics, Indianapolis, IN, USA), NEFA and cholesterol (Wako Chemicals, Osaka, Japan). Plasma human rASP levels were measured by an in-house ELISA method which does not cross-react with mouse ASP; background non-specific (PBS) values were subtracted. The final circulating recombinant ASP level was 8.0 nmol/L in both ND and HFD fed mice. Insulin levels were measured by ELISA (Crystal Chem Inc, Downers Grove, IL, USA). Mouse IL-1α, IL-1β, IL-2, IL-3, IL-4, IL-5, IL-6, IL-9, IL-10, IL-12(p40), IL-12(p70), IL-13, IL-17A, Eotaxin, G-CSF, GM-CSF, IFN-γ, KC, MCP-1, MIP-1α, MIP-1β, RANTES and TNF-α from adipose tissue or liver media were measured using suspension bead array immunoassay kits following manufacturer’s specifications (Bio-Plex Pro Mouse Cytokine Assay 23-plex, Bio-Rad, Mississauga, ON, Canada) on a Bio-Plex series 100 instrument (Bio-Rad, Mississauga, ON, Canada).

### Insulin Tolerance Tests

On day 15 post mini-pump insertion, an insulin tolerance test (ITT) was performed following a 6 h fast. Blood samples were taken at 0, 15, 30, 60, 90 and 120 min after an intra-peritoneal insulin injection (0.75 unit/kg of body weight). Glucose was measured using a glucometer, and insulin as described above.

### Tissue Lipids and Glycogen Measurement

Liver and muscle neutral lipids (TG and DG) and NEFA were extracted from tissue pieces in heptane: isopropanol (3∶2). The extract was transferred while the remaining tissue (liver and muscle) was air-dried, dissolved in 0.3N NaOH and assessed for protein content using the Bradford method (Bio-Rad, Mississauga, ON). Organic extracts were lyophilized and lipids were dissolved in 10% Triton X-100 aqueous solution. TG and NEFA were measured using commercial colorimetric kits as described above. Results are expressed as nmoles of TG or NEFA per mg of protein. Liver glycogen was measured as previously described [34]. Results are expressed as µmoles of glucose/g of liver weight.

### Lipolysis Assay

Inguinal adipose tissue was collected and dissected to removed blood vessels, then placed in room temperature, serum free-media, minced into small pieces (10–20 µg) and transferred into a 48-well plate. The tissue was treated with insulin (100 nM) or control (PBS) in 200 µl Ca^2+^-free Krebs–Ringer Buffer containing 1% fatty acid-free BSA and 5 mM glucose for 3 h at 37°C. NEFA and glycerol released were measured using colorimetric enzymatic commercial kits as described above. Soluble protein was extracted and measured as described above and the results are expressed as nmol/µg tissue protein.

### Adipocyte Histology

For adipocyte histological analysis, paraffin sections of inguinal and perirenal tissues (4 µm thick) were preserved in 4% paraformaldehyde and fixed onto slides using standard histological methods. Paraffin sections were stained with hematoxylin and eosin (Sigma Aldrich, Oakville, Ontario, Canada). Adipocyte area (300 cells/mouse tissue) was measured using Image-Pro Plus (Media Cybernetics, Bethesda, MD, USA) under 20X magnification (Olympus) in 3 random fields on a selected section per mouse and 9–10 mice per group were analyzed.

### Real-time Quantitative PCR (RT-qPCR)

Tissue mRNA was extracted, purified and reverse transcribed into cDNA using RNeasy Mini kits or RNeasy Lipid Tissue Mini kit and QuantiTect Reverse Transcription kits (Qiagen, Gaithersburg, MD). mRNA expression for GAPDH, C5L2, IRS1, GLUT4, GLUT2, SREBPF1, CD36, NF-κB, CCR2, FABP4, JNK were measured using QuantiTect Primer Assays (Qiagen, Gaithersburg, MD). mRNA for F4/80, CD11c and CD163 was quantified using custom primers (F4/80 forward: CTTTGGCTATGGGCTTCCAGTC reverse: GCAAGGAGGACAGAGTTTATCGTG, CD11c forward: CTGGATAGCCTTTCTTCTGCTG reverse: GCACACTGTGTCCGAACTC, CD163 forward: GGGTCATTCAGAGGCACACTG reverse: CTGGCTGTCCTGTCAAGGCT). Relative gene expression was calculated and corrected using GAPDH as the housekeeping gene. All procedures followed manufacturer’s instructions and MIQE guidelines (SABiosciences, Frederick, MD, USA and Bio-Rad Laboratories, Inc).

### Statistical Analysis

Results are expressed as Mean ± SEM. Groups were compared using Two-way ANOVA or Student t-test using Prism 5.0 software (GraphPad, San Diego, CA, USA). Statistical significance was set as p<0.05, where NS  =  not significant, *<0.05, **<0.01, ***<0.001.
